# A guide for comparing microbial co‐occurrence networks

**DOI:** 10.1002/imt2.71

**Published:** 2023-01-10

**Authors:** Chi Liu, Chaonan Li, Yanqiong Jiang, Raymond J. Zeng, Minjie Yao, Xiangzhen Li

**Affiliations:** ^1^ Engineering Research Center of Soil Remediation of Fujian Province University, College of Resources and Environment Fujian Agriculture and Forestry University Fuzhou China; ^2^ Key Laboratory of Environmental and Applied Microbiology, CAS, Environmental Microbiology Key Laboratory of Sichuan Province, Chengdu Institute of Biology Chinese Academy of Sciences Chengdu China

## Abstract

The article provides a pipeline for comparing microbial co‐occurrence networks based on the R microeco package and meconetcomp package. It has high flexibility and expansibility and can help users efficiently compare networks built from different groups of samples or different construction approaches.

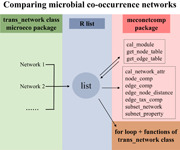

## INTRODUCTION

Microorganisms are ubiquitous in diverse environments of the earth and play important roles in ecosystem functions ranging from biogeochemical cycles [1] to the maintenance of host health [2, 3]. Microbial assemblages are generally comprised of a large number of species, which is represented as a “microbial community” defined in the context of different spatiotemporal scales. Identifying microbial members and their abundance in a community is a basic task in microbial ecology studies. Over the past two decades, a giant leap forward in sequencing techniques has made this task possible, leading to a rapid increase in data size. Furthermore, the advances of bioinformatic softwares (e.g., QIIME2 [4]) have profoundly improved the speed and convenience of sequence analysis. After obtaining operational taxonomic units (OTU), amplicon sequence variants (ASV) or species and their abundances (sequence counts or estimated abundance) in the bioinformatic analysis process, the following statistics and visualizations can be performed using R language [5] and related packages, which have grown up to be a cutting‐edge system in the recent decades [6]. Many statistical approaches in ecology can be used in microbiome data analysis benefiting from the similarities between macro‐ and micro‐ecosystems. However, there are also some dissimilarities in the research methods and routes between these two ecosystems [2]. The invisibility of microbes, a high proportion of uncultured species, and huge diversity in ecosystems lead to the difficulty of hypothesis‐driven studies, especially those referring to microbial interactions and functions. Researchers usually need to try a series of statistical tools and approaches and find the suitable one to verify a hypothesis. To maximize the accessibility of the R language and provide a good user experience, code must be organized in a manner that adheres to both conciseness and functionality. Based on this background, the R microeco package [7] was developed using the R6 class to make the customized pipeline easier and faster. In addition, the file2meco package [8] was also developed to facilitate the conversion of data files from other software, such as QIIME2 [4] and HUMAnN [9].

The co‐occurrence network analysis is often used to decipher the hidden patterns of complex microbial consortia in a wide range of spatiotemporal studies (see [10] and the references therein). In contrast to networks built for macro‐organisms (mainly based on the observations of species interactions [11, 12]), microbial co‐occurrence networks are mostly constructed from count tables obtained from amplicon or metagenomic sequencing data. The sequencing data have several general issues rendering network construction a challenge, including compositionality (sequence counts denote proportions instead of absolute abundances), sparsity (a large number of zeros), and difficulty for the inference of direct associations between paired taxa. Another challenge is how to explain the edges and their signs of the co‐occurrence network given that the edges are not recommended to be interpreted as microbial interactions for the cross‐sectional data [13]. To the best of our knowledge, the correlation‐based (Pearson or Spearman correlation) network may be the earliest and widely used approach in diverse studied habitats, such as soil [14]. To address the issues existed in the correlation network, a range of approaches have been developed, including Sparse Correlations for Compositional data (SparCC) [15], Compositionally Corrected by REnormalization and PErmutation (CCREPE) [16], and Correlation inference for Compositional data through Lasso (CCLasso) [17]. Further, several approaches based on graphical model are created to robustly infer the direct associations between taxa and optimize network structure. For example, SParse InversE Covariance Estimation for Ecological Association Inference (SPIEC‐EASI) [18] combines data transformations developed for compositional data and algorithms for sparse neighborhood and inverse covariance selection to reconstruct network. FlashWeave approach [19] adopts a local‐to‐global learning framework to infer the directly associated neighbors (i.e., Markov blanket) of a taxon and has good scalability and high speed on large heterogenous data sets. The studies on network comparisons [20] and approach reviews [10] have thoroughly discussed the robustness of different approaches and particularly recommended suitable network approaches depending upon different challenges. In addition, BEEM‐static method [21] is dedicated to seek out the interactions for cross‐sectional microbiome data with the generalized Lotka‐Volterra (gLV) model and an expectation‐maximization algorithm, offering a directed network to gain insight into microbial co‐occurrence in communities.

Along with the network approach development, some controversial voices worry about the misuse of network‐related methods (especially correlation network) on the inference of biotic interactions [13, 22, 23]. The main reason is that species interactions hold hub roles in answering many questions in community ecology. Another reason is that, no matter which network method is used, the edges can contain more or less information of species interactions depending on the approaches, parameters, and data features themselves. There is also another case that some actual species interactions may not be captured because of the approach itself or biological system characteristics (e.g., higher‐order biological interaction [24, 25]). Broadly speaking, the interpretations on how network edges represent have been largely overlooked mainly due to its difficulty. Although recently developed approaches are dedicated to reveal the direct interactions among microbes [19, 21], the applicability of these methods is still not clear when heterogeneous data sets are applied. So it is not recommended to explain co‐occurrence network as the interaction network [13, 26], producing a dilemma for users on how to explain the co‐occurrence network and to what extent the co‐occurrence network can represent the species interactions. Researchers have appealed to explain the inferred networks with biological background knowledge in previous reviews [13, 26]. Even in macroecology with empirical species interaction information, a study has revealed that associations among taxa rarely matched empirical net or direct species interactions [27], highlighting the inadvisable practice of coupling species co‐occurrences to species interactions. In terms of the ecological hypothesis, currently frequently used network analysis is largely different with other ecological approaches, such as null model and constrained ordination analysis. The direct or conditional associations among taxa represent the deterministic patterns in microbial consortia, as random assemblages (such as drift and dispersal in neutral process) cannot generate strong co‐occurrence patterns. So the network approach can be understood as a new application of the classic “checkerboard distribution” approach. The co‐occurrence patterns are derived from the joint species distribution and historical legacy. Various layers of complexity inherent to ecological systems (e.g., the variability of multiple abiotic factors and the interactions between abiotic and biotic factors) blur the inference that whether an association is a real interaction and how abiotic factors influence the association. Moreover, different network construction approaches correspond to dependent models in the underlying algorithms, also generating a problem in the data interpretation.

**Figure 1:**
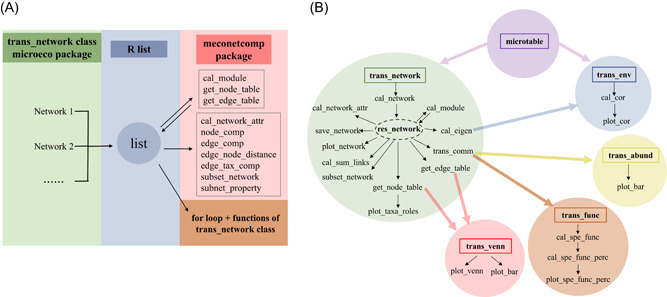
Schematic diagram of the approaches used in this protocol. (A) The schematic diagram of the whole protocol. (B) The flowchart comprised of the related classes and functions of microeco package in the protocol. The box denotes the class. The arrow represents the directed flow in the data analysis. The text without a box denotes the function in the corresponding class.

Although the prevalence of network approach has boosted a burst of research on various microbial fields, it is far from enough to provide an ecological hypotheses and explain the mechanisms behind the descriptive metrics. The methods implemented in software are generally a “black‐box” and lack the flexibility to combine the network analysis and other types of analysis methods. To reasonably use the network approaches, researchers need a thorough understanding of the analysis flows and the details of the network construction. In practice, it is important to combine multiple network approaches to improve the robustness of the edge finding. Moreover, in research with complicated experimental design, network construction may be performed for different treatments or groups [28], highlighting the urgency of the user‐friendly pipeline for network comparison. There are already several integrated online tools (e.g., MetagenoNets [29] and iNAP [30]) and R packages (e.g., igraph [31], NetCoMi [32], and ggClusterNet [33]) devoted to network analysis and visualizations. But the comparison of multiple networks is still a daunting challenge. In this protocol, to facilitate network comparison, R package meconetcomp (https://github.com/ChiLiubio/meconetcomp) was created (Figure 1A). The protocol mainly introduces the usage of the trans_network class of microeco packages and as much as possible combines other classes to show the power of the microeco package for network analysis (Figure 1B).

## METHOD

### Definition

A microbial co‐occurrence network is characterized as an edge‐weighted graph G = (V, E), where V (node) represents a feature (ASV/OTU/species) and E (edge) encodes the inferred connection in the network. The weight of E denotes the strength of the connection. The signs (+ or −) of edges represent the inferred positive or negative associations.

### Prerequisite

#### Install R and required packages

The user should first make sure the R language (https://cran.r-project.org/) [5] has been installed. Rstudio (https://posit.co/download/rstudio-desktop/) is highly recommended to be installed to facilitate the use of R language. R package meconetcomp (pronounced as [miːkəunetˈkʌmp]) was created to make the network comparison easier (Figure 1A). All the classes and functions of R microeco package used in the protocol were shown in the flowchart (Figure 1B).

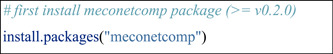

Then, several R packages deposited in CRAN (The Comprehensive R Archive Network) should also be installed.

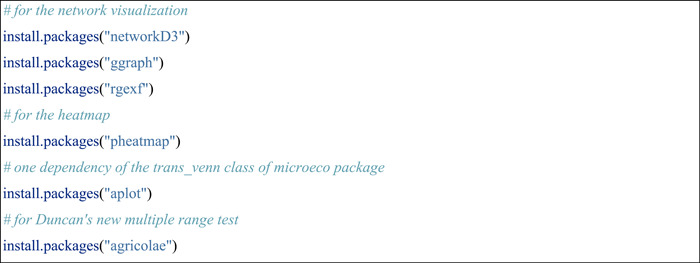

#### Prepare the data set

Two data sets inside the package meconetcomp were used in the protocol for different aims. One is the 16S rRNA gene amplicon data set published in the previous studies [8, 34], which has 200 soil samples from 48 sites of Chinese wetlands. This data set was used to compare networks created from different groups of samples. The other is the metagenomic data set of stool samples selected from R package ExperimentHub [35]. The data set has 92 species and 198 samples, collected from people with alcohol‐drinking habits. Using the metagenomic data can help us explore the co‐occurrence network at species level.

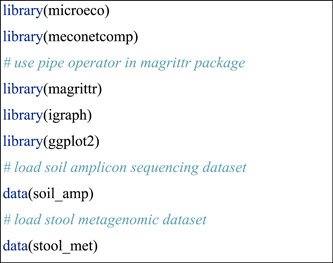

▲ Trouble shooting:

- The data set soil_amp and stool_met are both microtable objects that have been prepared. How to import the own data of users? Please read the help document and the tutorial of the microtable class of microeco package (https://chiliubio.github.io/microeco_tutorial/basic-class.html#microtable-class).

### Examples

#### Network construction across different groups

In microbial studies, it is common to construct multiple networks with different treatments or groups. Now we indicated an example of the operation of correlation‐based network construction across different groups using soil_amp data set. Correlation‐based network is designed to be constructed by accessing the res_cor_p list in the object, which is the return value after creating trans_network object. To perform network analysis, the first step is to create a trans_network object. If a correlation network is used, the user should assign a method to cor_method parameter to calculate correlation coefficients and p values. Then, network construction can be performed with the cal_network function. After using cal_network function, the res_network (i.e., the network with igraph class) is stored in the trans_network object as shown in Figure 1B. Here we created a list to store all the network objects for the following network analysis and comparisons with meconetcomp package (Figure 1A). Samples in the soil_amp data set are classified into three groups, Chinese inland wetlands (IW), coastal wetlands (CW) and Tibet plateau wetlands (TW), for the network analysis.

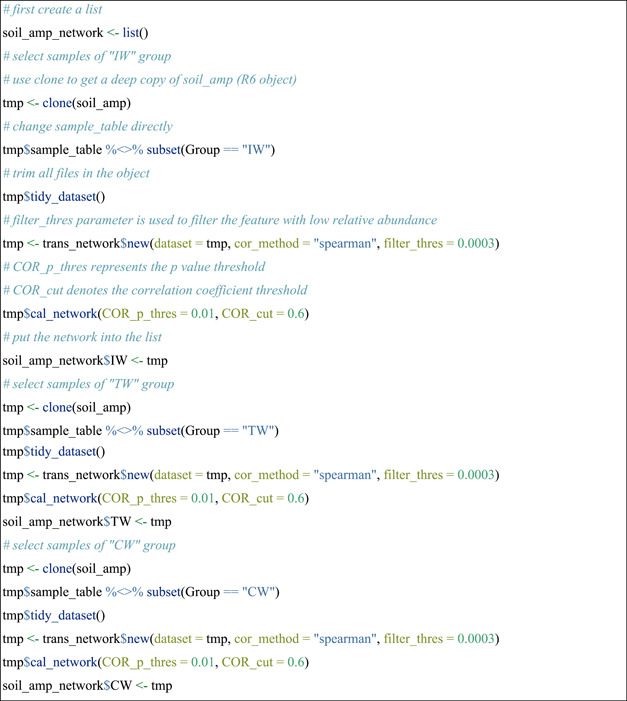

▲ Trouble shooting:

- For Pearson or Spearman correlation of large datasets, adding parameter “use_WGCNA_pearson_spearman = TRUE” can largely speed up the calculation. Note that using this parameter requires the WGCNA package to be installed (https://cran.r-project.org/web/packages/WGCNA/).

#### Network construction using different approaches

Another advantage of the above‐shown operation is that it can also be applied to the comparison of networks constructed from different approaches or different parameters of an approach. In this section, several network construction approaches were introduced step by step with the stool_met data set. For other network construction approaches not shown in this protocol (e.g., SpiecEasi [18], FlashWeave [19], and BEEM‐static [21]), please see the help document of the cal_network function of trans_network class in the microeco package.

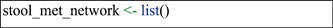

Create a network with Bray‐Curtis index (1—dissimilarity) between paired species.

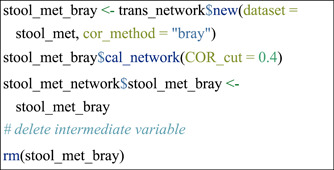

Then, networks were built using Pearson and Spearman correlations with different coefficient thresholds.

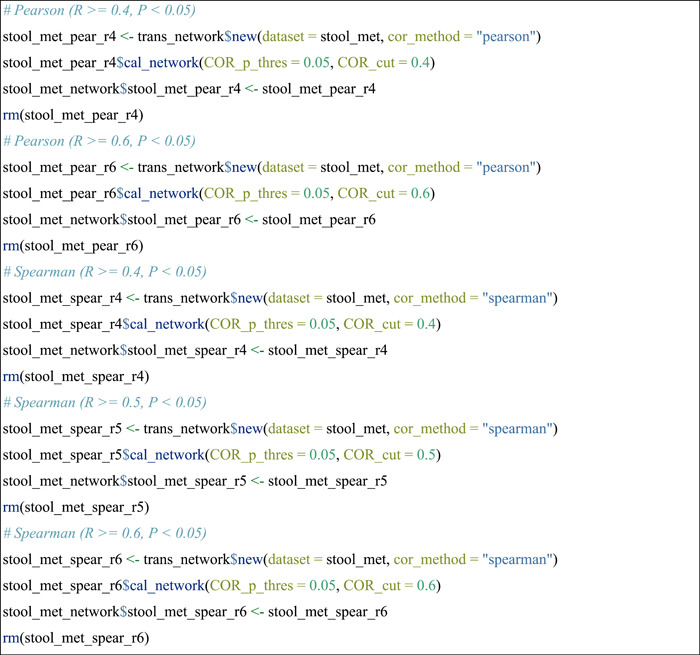

▲ Trouble shooting:

- For more parameters or approaches of network construction, please see the function cal_network of trans_network class with the command: help(trans_network).

#### Network modularity

Network modularity measures the strength of division of a network into modules (also called groups or clusters). Many algorithms based on igraph package have been encapsulated into cal_module function of trans_network class to partition the modules. After assigning modules to the network, the res_network in each trans_network object has the “module” label in the node attributes. Now we calculate the modules for all the networks in the list by using the function cal_module in meconetcomp package.

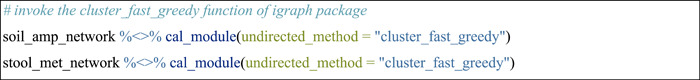

#### Network topological attributes

The global topological attributes of the network can be obtained by the function cal_network_attr in trans_network class. The return value of the function is res_network_attr table stored in the trans_network object. To compare the attributes across multiple networks, we extracted all the res_network_attr tables and merged them into one final table by using cal_network_attr function in meconetcomp package.

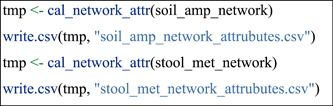

#### Node and edge properties

The function get_node_table and get_edge_table in trans_network class of the microeco package can be used to obtain a basic node property table (return value: res_node_table in the object) and an edge property table (return value: res_edge_table in the object), respectively. In get_node_table function, the node_roles parameter is designed to calculate within‐module connectivity and among‐module connectivity of nodes. Here, we indicated the examples of get_node_table and get_edge_table functions of meconetcomp package with pipe operators to directly perform the calculations for all the networks.

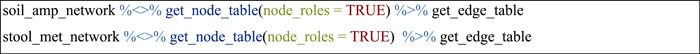

#### Network visualization

The trans_network class provides two types of network visualization. One is directly plotting the network in R. There are also two different plotting methods in R: static and dynamic network visualizations. The static visualization is suitable for the network with relatively less nodes number as the labels of nodes are prone to overlap. Currently, the implemented static approaches come from the functions of the igraph and ggraph packages.

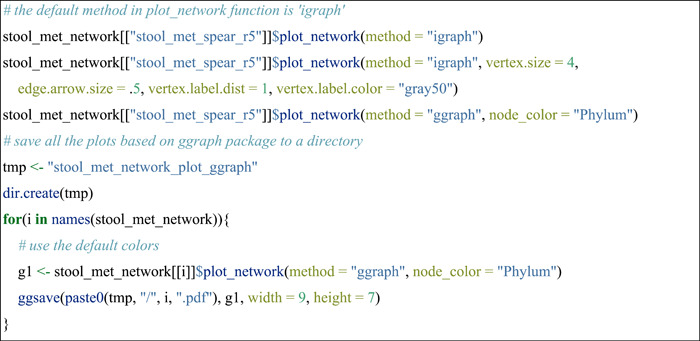

The dynamic visualization is developed based on the networkD3 package. This is especially recommended to take a glance at the network when a network has a large number of nodes and/or edges.

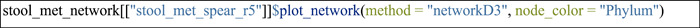

Another way is to save the network to the computer and perform the network visualization using Gephi software (https://gephi.org/).

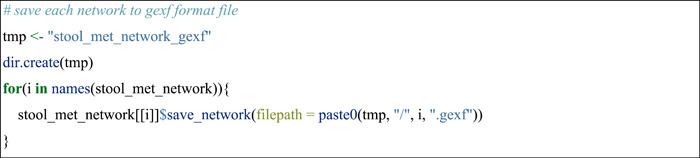

#### Compare nodes across networks

Comparing nodes and edges across networks is an attractive topic in network comparisons. The nodes in all the networks can be converted to a new microtable object by using the node_comp function of meconetcomp package. Then, it is easy to analyse the nodes overlap with trans_venn class (Figure 1B).

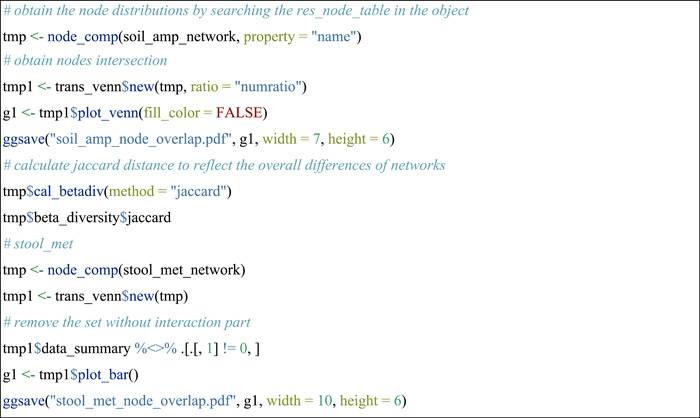

#### Compare edges across networks

The pipeline of studying edges overlap is similar to the above operations of nodes comparison. The edge_comp function of the meconetcomp package is used to convert edge distribution to a new microtable object.

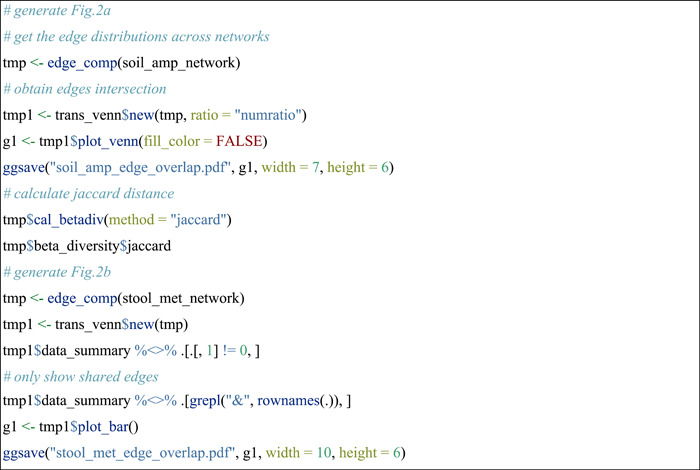

**Figure 2:**
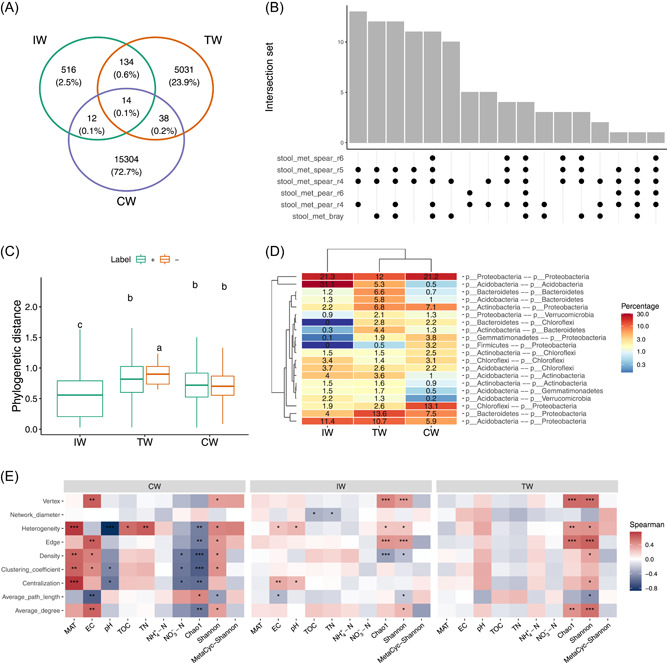
Visualization of a part of the analysis results in the protocol: (A) Venn diagram of edges between three networks in soil_amp_network. (B) The intersection set of edges of all the networks with different construction approaches in stool_met_network. (C) Phylogenetic distances between the nodes of the edges in soil_amp_network. Label “+” represents positive edges, that is, edges with positive associations. Label “−” denotes negative edges, that is, edges with negative associations. Different lowercase letters denote significant differences among different groups in analysis of variance analysis with Duncan's new multiple range test using duncan.test function of agricolae package (*p* < 0.05). (D) The number distribution of the phyla affiliated to the linked nodes of positive edges in networks of soil_amp_network. The number in the plot denotes the ratio of edges against all the positive edges in the network. (E) The correlations between subnetwork properties and abiotic factors/diversity. *p < 0.05, **p < 0.01; ***p < 0.001. CW, coastal wetlands; EC, electronic conductivity; IW, Chinese inland wetlands; MAT, mean annual temperature; MetaCyc–Shannon, the Shannon entropy based on the MetaCyc pathway abundance of metagenomic sequencing of wetland soils; TN, total nitrogen; TOC, total organic carbon; TW, Tibet plateau wetlands.

#### Extract overlapped edges of networks to a new network

Then we extracted the subset of edges according to the intersections of edges across networks, which can be accomplished with the subset_network function in meconetcomp package.

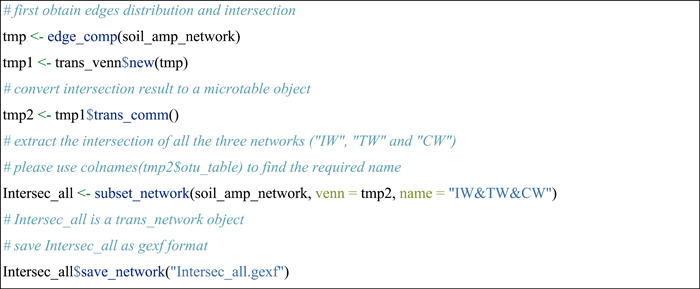

#### Compare phylogenetic distances of paired nodes in edges

The edge_node_distance class (R6 class) in the meconetcomp package is developed to compare the distribution of distance values of paired nodes in all the edges across networks. Here, we indicated the phylogenetic distance distributions and performed the differential test among networks (Figure 2C). The input parameter dis_matrix can be any symmetric matrix with both the column names and row names (i.e., feature names). So it is also feasible to compare other properties of features, such as Levin's niche overlap [36].

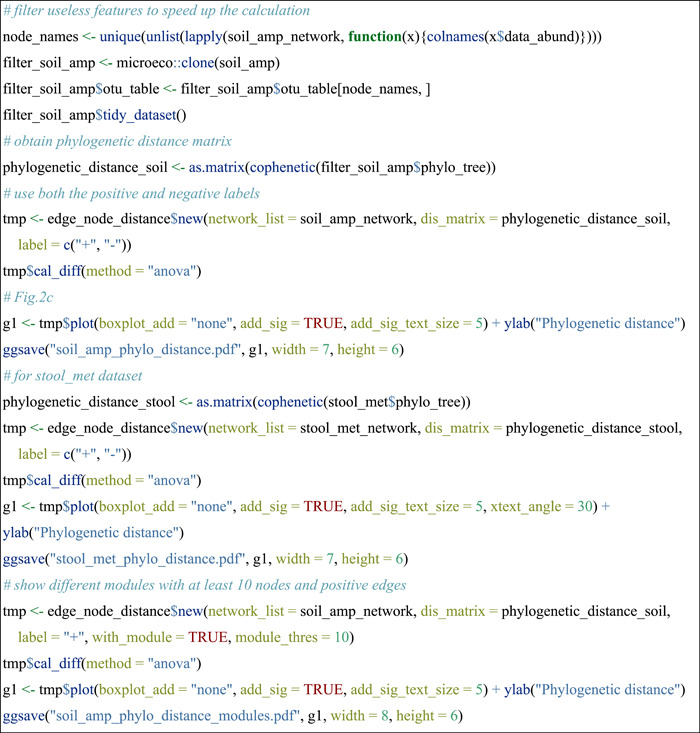

#### Node topological roles

Then, the node classification and visualization based on the within‐module connectivity (Zi) and among‐module connectivity (Pi) were performed. Nodes were classified into four categories [7, 37]:

(1) Module hubs (nodes with highly links within their own module, Zi > 2.5 and Pi ≤ 0.62); (2) Connectors (nodes that connect different modules, Zi ≤ 2.5 and Pi > 0.62); (3) Network hubs (nodes that act as both module hubs and connectors, Zi > 2.5 and Pi > 0.62); (4) Peripherals (nodes that have only a few links and almost always to the nodes within their own modules, Zi ≤  2.5 and Pi < 0.62).

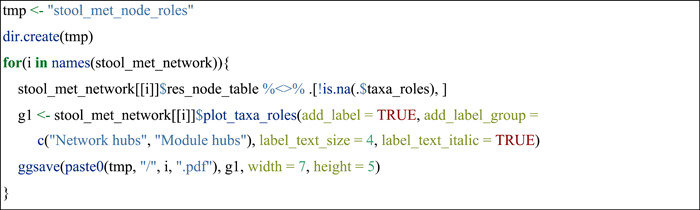

#### Compare node sources of edges across networks

To know which taxa constitute the nodes in edges is important in understanding species co‐occurrence patterns and answering ecological questions. In this part, as an instance, we used edge_tax_comp function of the meconetcomp package to get the sums of node sources (at Phylum level) in the positive edges. In other words, how many linked nodes of positive edges come from different phyla or the same phyla. Then, to make the results comparable, the ratio was calculated with the positive edge number as denominator.

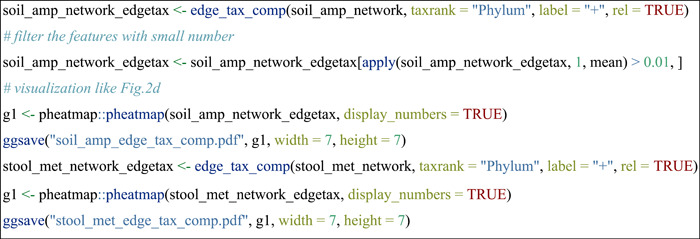

#### Module eigengene analysis

To assess how network modules emerge along with different effects of abiotic factors, the module eigengene analysis was performed using the cal_eigen function in trans_network class. Then the correlation analysis between module eigengenes and abiotic factors was applied to infer the factors influencing different modules. This part was shown with the soil_amp data set.

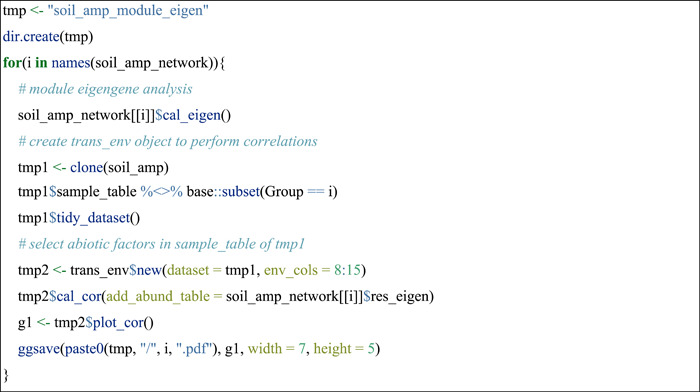

#### Taxonomic compositions and functional predictions in different modules

The function trans_comm in trans_network class is designed to convert classified nodes (i.e., the group information in res_node_table) directly to community‐like microtable object. We showed the OTU compositions of modules at Phylum level and functional predictions with FAPROTAX database [38] using soil_amp data set.

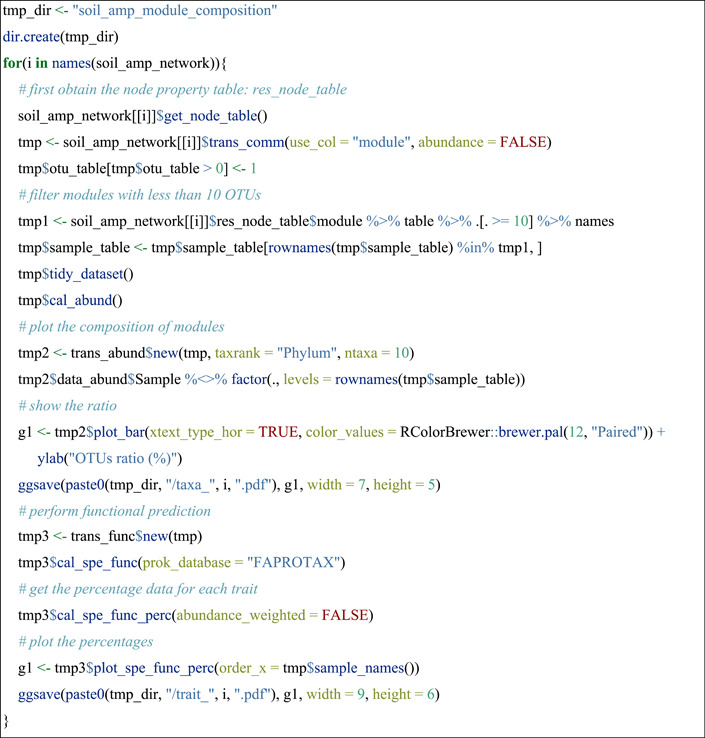

#### Compare topological properties of subnetworks

In this part, we extracted the subnetworks according to the OTU existing in each sample of soil_amp data set for each network in soil_amp_network. Then, the global topological properties of subnetworks were calculated. All the operations were encapsulated into the subnet_property function of the meconetcomp package.

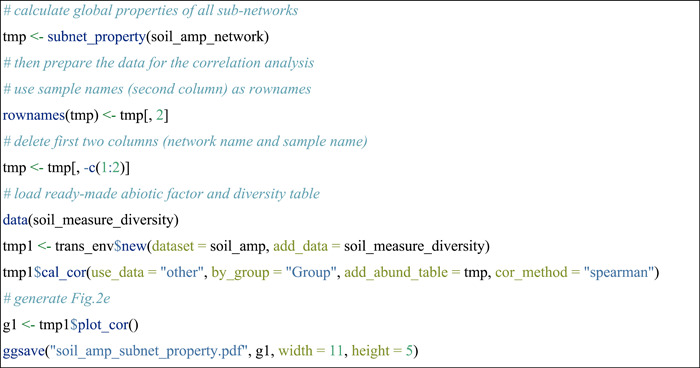

## DISCUSSION

### High flexibility and expansibility of the current protocol in network comparison

In this protocol, we mainly introduced the pipeline for the analysis and comparisons of multiple co‐occurrence networks constructed from different groups of samples or different network approaches. Different from the network comparison approach in NetCoMi package [32], we take full advantage of the R list class, trans_network class of microeco package and the meconetcomp package to perform each part of network analysis. The most noteworthy advantage is the number of networks for comparison has no limitation in the practice. Furthermore, the pipeline has high flexibility and expansibility to be applied in other network analysis not covered in this protocol. To date, no standards for network approach selection are available in microbial ecology. Nor is there a “one size fits all” tool that can automatically match a set of network methods or parameters for the user's data set. Similar to the results from a benchmark study that correlation detection strategies in microbial data sets vary widely in sensitivity and precision [20], we also found the results across network methods vary at each aspect (Figure 2B and other saved figures using the stool_met_network data set). We argued that, except for the empirical selections of network approaches [10, 20, 25], the user's comparison on the network approaches is also feasible and valuable to learn more on what network approach can reflect and explain by integrating other ecological and statistical methods (such as null model) implemented in the microeco package and other R packages.

### Linking network results to ecological patterns and biological background

Disentangling the environmental effects on microbial association networks is another attractive topic [39]. Yet the lack of gold standard data may diminish power and feasibility when studying these questions. A recent report shows that rare positive interactions are likely caused by metabolic cross‐feeding among microbes in soil [40]. So in heterogeneous habitats like soil, co‐occurrence network results should be directed more to the ecological patterns instead of the interpretation of how species interacts. Even though the lack of standard data set with determinate species interactions and ecological patterns has largely impede methodological development and limit our ability to broadly link network results to ecological patterns, it is still feasible to compare different networks built from different groups of samples to reveal the ecological patterns and infer the assembly processes by combining network method and other ecological approaches. For instance, differential phylogenetic distance (Figure 2C) across different groups in soil_amp data set showed that taxa associations (strong positive correlations) might be caused by different abiotic factors and varying extent of deterministic processes. Furthermore, different factors might affect the topological structure of networks in the soils from different categories of Chinese wetlands (Figure 2E). With the increasing number of cultured species and metagenome‐assembled genomes, it is possible to verify the biological meaning of the shared edges (Figure 2B) among different network approaches, and figure out how and to what extent the paired species interact [41] in the studies with important biological mechanisms in explaining the results. While a full analysis of all the network related approaches is beyond the scope of this study [28], these examples in the protocol demonstrated the power of the pipeline mainly generated by the combination of the R list, “for” loop, trans_network class and the meconetcomp package. To make the pipeline of the protocol easy to use, we did not consider too many factors on data manipulations, such as rarefaction, sparse data filtering, and network construction parameters. These factors and operations may be critical in customized data analysis in the sense that they can affect the robustness of results expected. In addition, since the R6 object has the special attribute of binding functions to the objects, the loop approach in this protocol can also be applied in other data analysis with microeco package, such as comparisons of different groups of samples in other classes.

## AUTHOR CONTRIBUTIONS

All authors contributed to the protocol development. The initial idea was conceived by Chi Liu, Minjie Yao, and Xiangzhen Li. During the protocol development, all the authors contributed to the test and improvements. The original manuscript was written by Chi Liu, Minjie Yao, and Xiangzhen Li, and revised by Chaonan Li, Yanqiong Jiang, and Raymond J. Zeng.

## CONFLICT OF INTEREST

The authors declare no conflict of interest.

## Data Availability

Besides the datasets loaded from meconetcomp package, the data sets used in the protocol are also deposited in GitHub (https://github.com/ChiLiubio/network_protocol) and Gitee (https://gitee.com/chiliubio/network_protocol). Supporting Information materials (figures, tables, scripts, graphical abstracts, slides, videos, Chinese translated versions, and update materials) may be found in the online DOI or iMeta Science http://www.imeta.science/.
